# 821 11-Year Experience in Management of Genital Burns

**DOI:** 10.1093/jbcr/iraf019.352

**Published:** 2025-04-01

**Authors:** Mare Kaulakis, José Arellano, Christopher Fedor, Hilary Liu, Garth Elias, Paul Rusilko, Alain Corcos, Jenny Ziembicki, Francesco Egro

**Affiliations:** University of Pittsburgh School of Medicine; University of Pittsburgh Medical Center; University of Pittsburgh Medical Center; University of Pittsburgh Medical Center; University of Pittsburgh Medical Center; University of Pittsburgh Medical Center; University of Pittsburgh Medical Center; University of Pittsburgh Medical Center; University of Pittsburgh Medical Center

## Abstract

**Introduction:**

Genital burns pose unique challenges due to their anatomical location, complexity, and infection risk. Despite affecting only 1% of total body surface area (TBSA), these burns are associated with a high mortality risk. Treatment protocols vary, and standardized approaches are still needed, particularly for full-thickness burns. This study aims to develop an algorithm for genital burn management based on a single institution’s 11-year experience.

**Methods:**

A retrospective analysis was conducted on patients presenting to a single ABA-verified burn center from 2012-2023 with genital burns. Data collected included demographics, burn characteristics, treatment strategies, and outcomes.

**Results:**

62 patients (69% male, 31% female; mean age of 31.13 ± 26.20 years) were included in the study. Average BMI was 26.94 ± 12.4, with the most common comorbidities being hypertension (27.42%; n=17) and smoking (16.13%; n=10). Most burn etiologies were scald (45.2%; n=28) and flame (38.7%; n=24), and the mean %TBSA was 18.04 ± 18.72. Almost all 29 of superficial and superficial partial thickness burns were managed conservatively (96.55%; n=28). Meanwhile, 17 of the 33 deep partial thickness and full thickness burns required surgical intervention (51.52%). The most common procedures in patients receiving surgery were excision plus the application of a split thickness skin graft (STSG) (35.29%; n=6), or a two-stage operation involving excision plus cadaveric allograft, followed by STSG application at a later date (29.41%; n=5).

The mean number of total acute operations increased with increased burn depth. Patients with full thickness genital burns were 60.39 times (95% CI: 4.80-760.46; p=0.002) more likely to require surgery in this region compared to those with more superficial burns, and 9.78 times (95% CI: 1.13-84.35; p=0.038) more likely to undergo multiple surgeries compared to patients with more superficial burns who also received surgery.

Ultimately, 11.29% (n=7) of all patients had hypertrophic scarring and 17.65% (n=3) of surgical patients had graft loss. No patients experienced post-burn scar contractures. The average length of follow up was 1.99 ± 4.87 months.

**Conclusions:**

Genital burns present significant challenges and mortality risks. This study shows that deeper genital burn injuries are not only more likely to require surgical intervention but also require multiple operations. Based on these findings, an algorithm was developed, recommending that superficial genital burns be managed conservatively, while burns extending beyond the dermis are strong candidates for surgical intervention.

**Applicability of Research to Practice:**

This study contributes valuable insights into genital burn management, highlighting the necessity for tailored treatment approaches based on burn depth/location. The developed algorithm provides a structured framework that can guide clinical decision-making and improve outcomes in patients with these complex injuries.

**Funding for the Study:**

N/A

